# Dataset for facilitating the calculation of aspect ratio of fibrillated cellulose suspensions based on gel point data

**DOI:** 10.1016/j.dib.2023.109944

**Published:** 2023-12-18

**Authors:** J.L. Sanchez-Salvador, M.C. Monte, C. Negro, W. Batchelor, G. Garnier, A. Blanco

**Affiliations:** a Chemical Engineering and Materials Department, Universidad Complutense de Madrid, Avda. Complutense s/n, Madrid 28040 Spain; b BioPRIA, Chemical Engineering Department, Monash University, Melbourne, VIC 3800, Australia

**Keywords:** Nanofibrillated cellulose, Cellulose microfibers, Nanocellulose, Gel point methodology, Aspect ratio determination

## Abstract

This article describes data related to the research paper “Simplification of gel point characterization of cellulose nano and microfiber suspensions” [1]. The characterization of fibrillated celluloses that include cellulose nano and microfibrils (CMNFs) is a challenge for their production on an industrial scale, requiring easy techniques that control their quality and reproducibility. Gel point is a convenient parameter commonly used to estimate the aspect ratio (AR) of CMNFs. However, this estimation requires many sedimentation experiments, which are tedious and time consuming. The dataset includes all information related to the traditional experiments and to the simplified experiments for estimating gel point and AR based on only one sedimentation experiment. The full data set is useful to select the initial concentration to carry out the experimentation. This dataset also includes the information for the validation of the proposed simplified methodology and shows that the errors are lower than 7% for the gel point calculation and of 3% for the AR estimation. A larger databased of nanocellulose suspensions can be built with the reuse of this data to allow the estimation of nanocellulose properties in a future.

## Graphical abstract

**Figure:**
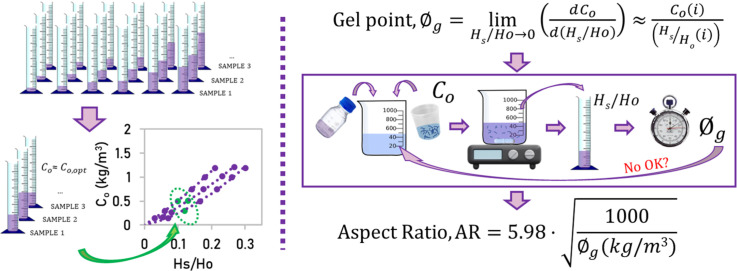

Specifications Table

**Table:** 

Subject	Materials Science
Specific subject area	Characterization of cellulose micro and nanomaterials
Data format	Raw, Analyzed, Filtered
Type of data	Table, Figure
Data collection	Data related to sedimentation experiments were collected by hand measuring the height of the fibrillated cellulose suspension versus time at different initial concentrations. Suspensions were selected to cover a wide group of fibrillated cellulose with very different properties and fibrillation degrees. The data were analysed by using a MATLAB Script to calculate the optimal fits of the gel point and using excel to calculate the aspect ratio and the gel point estimations.
Data source location	Universidad Complutense de Madrid, Madrid, Spain Monash University, Melbourne, Australia
Data accessibility	Repository name: Mendeley Data Data identification number: doi:10.17632/s7r9mrb24g3.1 Direct URL to data: https://data.mendeley.com/datasets/s79mrb24g3/1
Related research article	J.L. Sanchez-Salvador, M.C. Monte, C. Negro, W. Batchelor, G. Garnier, A. Blanco, Simplification of gel point characterization of cellulose nano and microfiber suspensions, Cellulose (2021) 28:6995–7006. https://doi.org/10.1007/s10570–021–04003–5.

## 1 Value of the Data

The aspect ratio (AR) of fibrillated cellulose materials, which encompasses cellulose fibers in different scales including cellulose nano and microfibrils (CMNFs), is a complicated data to be calculated from microscopy images [2]. The length of the fibers is difficult to measure in the images due to the entanglement of fibrillated cellulose forming a three-dimensional network and to the high length/diameter ratio of these materials [3].

An alternative method is the use of the gel point (Ø_g_), defined as the lowest concentration at which all fibers are interconnected in a suspension, forming a self-supporting network [4]. This technique has been previously used for the estimation of the AR in different cellulose materials and CMNFs, but it is very tedious [5], [6], [7], [8]. It requires several sedimentation experiments, which are time consuming when several fibrillated celluloses need to be characterized [1].

• These data are of value to the scientific community because they provide a simpler and faster method to estimate Ø_g_, and from this value, the AR and the average length of CMNFs.
• These data provide the information about the experimentation done for the simplification of the gel point methodology and the AR estimation based on a significant reduction of the sedimentation experiments required.
• These data include the validation of the simplification by using different fibrillated cellulose samples.
• The data of the different fibrillated celluloses can be reused individually to evaluate the differences in sedimentation and also to improve the range of AR in which the simplification is valid.

## 2 Data Description

The dataset is provided as an Excel file (DataGelPointUCM-MU.xlsx) including six sheets.

Sheet1 “MATLAB Script” is the script developed to calculate the gel point using the CSAPS function of MATLAB which requires the optimization of the “p” parameter as it is explained in methods. In this script, it is described the points in which the values of sedimentation (initial concentration (C_o_) and relative sedimentation height) must be included and also the parameters to optimize the “p” parameter which is not a fix value, and its selection depend on the value of Ø_g_.

Sheet2 “Co and Hs/Ho of 25 samples” include the raw data used to develop the simplification of the gel point methodology. 25 different raw materials have been used for this purpose using at least 4 sedimentation experiments at different C_o_.

Example1 “Calculation of Co” includes an example to calculate the C_o_ of a fibrillated cellulose using the quadratic fit. Then, with the minimum upper experimental error (UEE) is possible to calculate the estimated Ø_g_ at the Co and the error related to the Ø_g_ using different fits in the CSAPS function (Sheet1).

Sheet3 “Smooth spline” includes the Ø_g_ for the 25 fibrillated celluloses and the seven fits. In addition, the error between the Ø_g_ and the estimated Ø_g_ in the C_o_ in CSAPS is calculated, selecting which is the best one and the correlations between Ø_g_ and C_o._

Sheet4 “Aspect ratio” includes the AR calculated for the 25 fibrillated celluloses using the estimation in the C_o, opt_ and the best CSAPS fit.

Sheet5 “Validation” includes the data of the sedimentation experiments for the validation of the optimal interval of C_o_.

## 3 Experimental Design, Materials and Methods

### 3.1 Mathematical simplification

According to Martinez et al. (2001), the gel point can be obtained from the slope near the ordinate axis of the curve that relates the initial concentration of a fibrillated cellulose suspension and the relative height between the sediment and the total height (Hs/Ho), as Eq. (1) shows [8]. This curve must be graphed when the samples are completed settle and not depend on the time. Currently, two mathematical methods have been used for the calculation of the gel point: first, the quadratic fit without independent term in which the first degree coefficient is the gel point value; and second, the CSAPS fitting tool in MATLAB that use a smoothing spline with requires the introduction of a smoothing parameter (p) that must be optimized to avoid local fluctuations or and y-intercept far to zero [1].

(1) $$\;Gel\;point,\;\emptyset_{g\;}=\lim_{H_{s}\;/\;H_{o}\to 0}\left(\frac{dC_{o}}{d\left(H_{s}\;/\;H_{o}\right)}\right)$$

The assumption to simplify this methodology requires the replacement of the derivative by an increment between two concentrations (Eq. (2)). Since the gel point is calculated near the relative height close to zero, the increment is selected between a determined concentration Co(i) and a theoretical concentration of zero C_o_(0) with a relative height (Hs/Ho(0)) of zero. Therefore, the derivative is approximated as the quotient between the Co(i) and the relative sedimentation height (Hs/Ho(i)).

(2) $$\emptyset_{g}\approx \frac{C_{o}\left(i\right)-C_{o}\left(0\right)}{\left(H_{s}\;/\;H_{o}\left(i\right)\right)-\left(H_{s}\;/\;H_{o}\left(0\right)\right)}=\frac{C_{o}\left(i\right)}{\left(Hs\;/\;Ho\left(i\right)\right)\;}$$

### 3.2 Materials and experimental method

To characterize the Ø_g_ and the AR of a fibrillated cellulose material with a diameter fiber in the micro or nanoscale the materials required are the following:

• CMNFs at initial concentration about 1 wt.%
• Crystal violet at 0.1 wt.%
• Deionized water
• 250 mL graduated cylinders
• Magnetic stirrer

To prepare the CMNF suspension that allow the calculation of the Ø_g_ at a certain C_o_, an amount of CMNF gel is weighted and diluted in deionized water up to 250–260 mL. 200 µL of 0.1 wt.% crystal violet, that do not affect in the sedimentation of the sample, is added to dye the fibrillated suspensions. The suspension is stirred using magnetic agitation until no clusters were observed and avoiding intense agitations that break the fibers. 250 mL of the suspension are added into a graduated cylinder and settled until the sediment reach a steady value to obtain the complete deposition of fibrillated cellulose. This methodology is the same in the case of the traditional Ø_g_ using several sedimentation experiments (and the CSAPS or quadratic fit to obtain the gel point) or for the simplified one with required only one experiment in which Ø_g_ is calculated using Eq. (2).

A total of 25 CMNF samples have been used to study the assumptions to simplify this methodology. Data of most of them have been used in previous publications for other purposes. Their manufacturing methods are briefly summarized below in Table 1.

**Table 1:** List of raw materials and treatments to produce the CMNFs used in this study.

No.	Raw Material	Pretratment	Main Treatment	Reference and Comments
1–5	Recycled paper from newsprint	TEMPO-mediated oxidation (5 mmol NaClO/g pulp)	High pressure homogenization (HPH) 600 bar, 4 passes	[9] Stirring agitation of CMNFs: 3, 70, 125, 500, 2500 s ^−^ ^1^
6–9	Recycled paper from newsprint	Refining PFI mill 5 K rpm	HPH 600 bar, 6 passes	[9] Stirring agitation of CMNFs: 50, 500, 900, 2500 s ^−^ ^1^
10–13	Recycled bleached de-inked pulp	–	Refining PFI mill 0, 10 K, 30 K, 50 K rpm	[5]
14–16	Triodia pungens grass (spinifex) plants	Hot water + NaOH, Delignification with NaClO_2_	No HPH, HPH 1 pass 300 bar and HPH 1 pass 500 bar	[10]
17–20	Bleached Eucalyptus Kraft Pulp	–	Refining PFI mill 0, 10 K, 30 K, 50 K rpm	[5]
21–22	Bleached Eucalyptus Kraft Pulp	Refining PFI mill 10 K rpm	HPH at 500 and 1000 bar 1 pass	[10]
23	Bleached Eucalyptus Kraft Pulp	TEMPO-mediated oxidation (5 mmol NaClO/g pulp)	High pressure homogenization (HPH) 600 bar, 4 passes	Not published
24	Thermomechanical pulp	Water disintegration	Brecht-Holl 200 mesh screener (Fines <76 µm)	Not published
25	Kraft	Water disintegration	Brecht-Holl 200 mesh screener (Fines <76 µm)	Not published

### 3.3 Selection of the optimal smoothing spline in the CSAPS fit of MATLAB

**Fig. 1:**
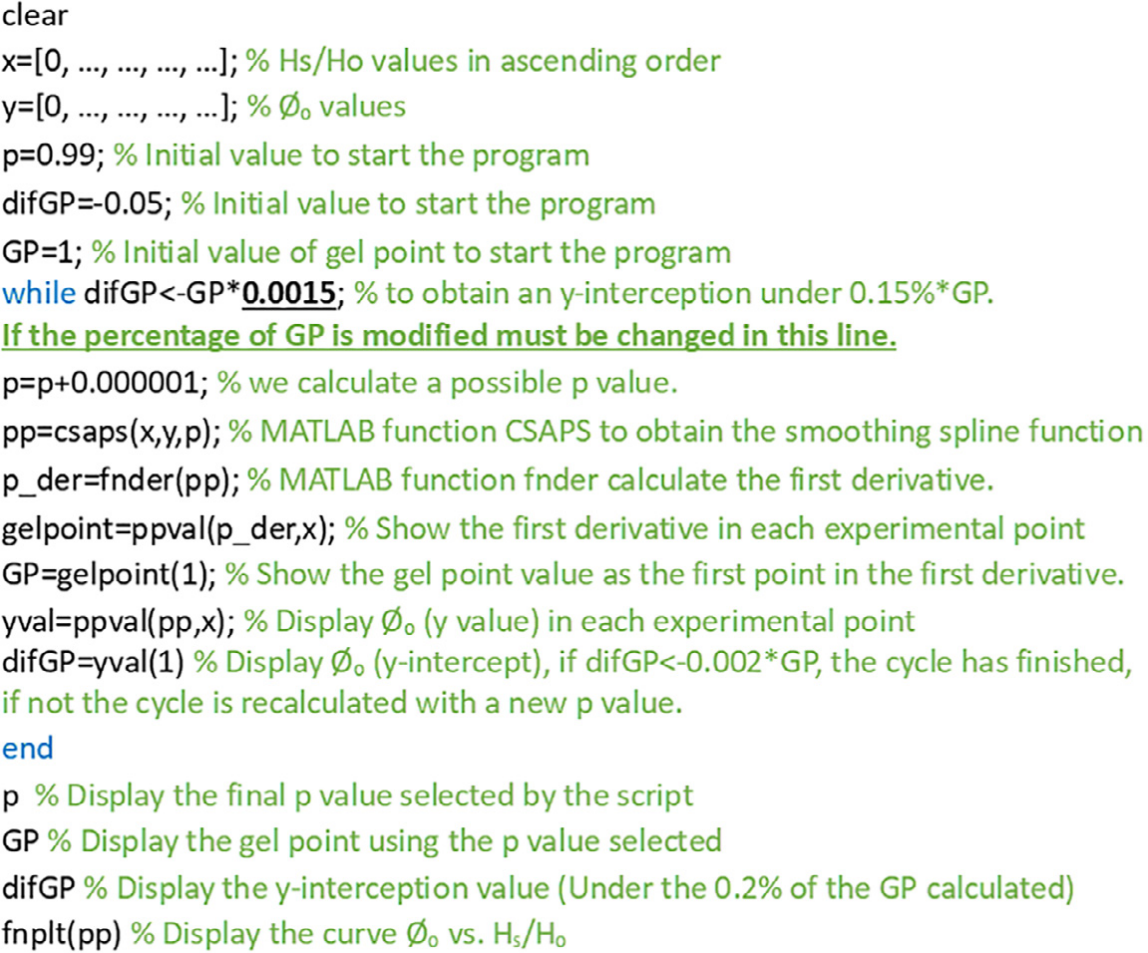
Script to obtain Øg according to a determined (y-interception < percentage·100·Øg). In the script shown the selection of the y-interception was under 0.15% of Øg (y-interception < 0.0015·Øg), that would be the optimal value.

To select the optimal smoothing spline, first of all, the “p” parameter” must be optimized for first time. This parameter “p”, ranging from 0 to 1, must be adequately chosen. Low “p” parameter values show in the graph Co vs. Hs/Ho a y-intercept far from zero and a linear trend that falsifies the Ø_g_, whereas a too high “p” parameter value shows a y-intercept close to zero but with local fluctuations (the graph try to connect the experimental values without obtaining a second order curve) [1]. Therefore, the optimal parameter is an intermediate “p” value” which is not a fix value and differs with the Ø_g_. For this reason, a MATLAB script (Fig. 1, Sheet1 of Excel Data Base) was developed to study the best p-value to obtain a low error (respect to the origin) in the y-intercept of the graph Co vs. Hs/Ho, but at the same time there are no local fluctuations. 25 fibrillated celluloses (traditional sedimentation values shown in Sheet 2 of Excel Data Base) were studied using the traditional sedimentation curves to evaluate the best fit in CSAPS of MATLAB.

The script used to choose the “p” parameter select a maximum in the y-intercept of the curve Co vs. Hs/Ho that is a percentage of the final Ø_g_ chosen (y-interception < percentage·100· Øg). Seven percentages were chosen (y-intercept <0.0005·Ø_g_; <0.0010·Ø_g_; <0.0015·Ø_g_; <0.0017·Ø_g_; <0.0020·Ø_g_; <0.0025·Ø_g_; < 0.0050·Ø_g_). For each percentage (7) and each fibrillated cellulose (25), the Ø_g_ is obtained (175 values).

**Fig. 2:**
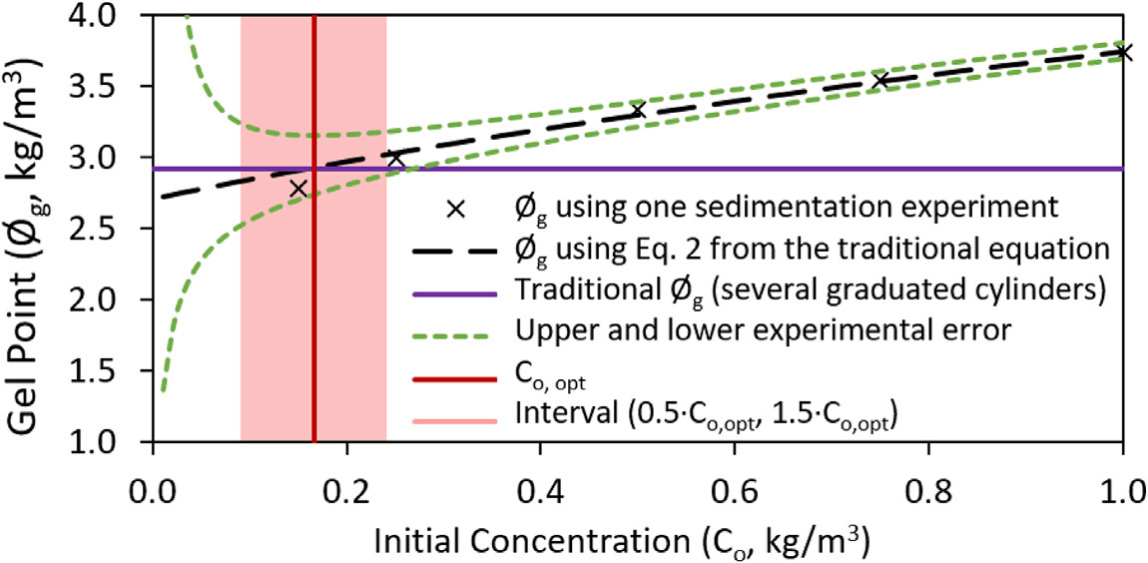
Estimation of gel point values (Ø_g_) vs. Initial concentration selected (C_o_). Comparison of traditional and simplified methods and experimental error limitations.

To evaluate the best “p” parameter condition, the Ø_g_ obtained with each “p” condition where compared with the optimal initial concentration (C_o,opt_). As Fig. 2 shows, the C_o,opt_ is selected in the point in which the upper experimental error (UEE) is minimum (UEE is represented with the dashed green line of Fig. 2). Example 1 of Excel data base shows an example to calculate C_o,opt._ To obtain this UEE due to the visualization of the settle, we assume an estimated error of Hs±1 mL. Ø_g_ in the UEE is recalculated using Eq. (2) but replacing Hs by Hs±1. The C_o,opt._ is selected in the point in which the Ø_g_ in the UEE is the minimum value.

In the Example 1 of Excel data, for this C_o,opt._ the estimated Ø_g_ was compared with the gel point traditionally calculated with several sedimentation experiments for two different conditions of “p parameter”. The first condition to obtain the “p” parameter was a Ø_g_ with a y-intercept < |0.0015·Øg|and another with a y-intercept < |0.005·Øg|. In the first case, we obtain a Ø_g_ error of 3.3%, whereas in the second case the error increase up to 7.7%.

Sheet 3 of Excel data base shows for the 25 fibrillated celluloses the estimated Ø_g_ in the C_o,opt_ and the Ø_g_ using seven conditions of “p” parameter. Table 2 indicates the linear fit between C_o,opt_ and Ø_g_ for each percentage used to choose the “p” parameter. The best correlation to obtain the “p” parameter is when the y-interception is selected under 0.15% of the Ø_g_ due to the lower relative error and the best correlation.

**Table 2:** Linear fit between C_o, opt_ and Ø_g_ for each percentage used to choose the “p” parameter.

Smooth spline	Correlations	R_2_	Relative Error (Average 25 samples)
*y-interception <0.0005·Øg*	*C_o,opt_ = 0.0578Ø_g_ – 0.0071*	*0.9666*	*5.6 %*
*y-interception <0.0010·Øg*	*C_o,opt_ = 0.0571Ø_g_ – 0.0096*	*0.9644*	*2.0 %*
***y-interception <0.0015·Øg***	*C_o,opt_****= 0.0572Ø_g_ – 0.0127***	***0.9650***	***0.1 %***
*y-interception <0.0017·Øg*	*C_o,opt_ = 0.0573Ø_g_ – 0.0146*	*0.9644*	*−1.2 %*
*y-interception <0.0020·Øg*	*C_o,opt_ = 0.0576Ø_g_ – 0.0172*	*0.9623*	*−2.3 %*
*y-interception <0.0025·Øg*	*C_o,opt_ = 0.0574Ø_g_ – 0.0196*	*0.9634*	*−4.1 %*
*y-interception <0.0050·Øg*	*C_o,opt_ = 0.0577Ø_g_ – 0.0324*	*0.9590*	*−10.7 %*

The most important point in this simplification is the selection of the C_o_, that is a key factor to obtain an adequate estimation of Ø_g_. The selection of the C_o, opt_ depends on several factors as the raw material, the pretreatments or the intensity of the mechanical treatments. Therefore, in the gel point simplification the initial C_o_ is an arbitrary value that has to be selected based mainly on the experience with other similar fibrillated cellulose or according to some recommendations based on the pretreatment and main mechanical treatments employed to obtain the fibrillated cellulose as Table 3 shows. Therefore, once selecting the C_o_ and calculated the estimated Ø_g_, it is possible to calculate, with the optimal equation of Table 2, the C_o, opt_ and compared it with the C_o_.

**Table 3:** Recommendations in the selection of the initial concentration (C_o_).

Pretreatments	Recommendations
Refining	0.05–0.5 kg/m^3^, using lower Co values as refining and main mechanical treatment intensities increases.
Enzymatic	0.2–0.5 kg/m^3^ AR is very similar after this pretreatment and after the main mechanical treatments of fibrillation.
Powder	Co >1 kg/m^3^. The decrease in length produces lower AR and higher Øg.
TEMPO-mediated oxidation	0.5–3 kg/m^3^, using higher Co values as the oxidant dose increases and as mechanical treatment intensity increases

In the case the values of C_o_ and C_o, opt_ are too different, where C_o_ is outside of the interval (0.5·C_o, opt,_ 1.5·C_o, opt_), a second sedimentation experiment is required using a new C_o_ closer to C_o, opt,_ repeating the above steps again. According to the method validation, in the cited interval of C_o, opt_ the Ø_g_ error is under 7% in the fibrillated celluloses used to validate this method.

From the gel point value and assuming a density of the cellulose of 1500 kg/m^3^, it is possible to calculate the average AR of the CMNFs according to the Crowding Number (CN) and to the Effective Medium Theory (EMT) using Eq. (3) and Eq. (4), respectively [2,^11^,12]. This information is included in Sheet4 of Excel data base. For this determination, the error in the same interval of Co, opt is under 3% for the samples used to validate the methodology. Finally, with the value of AR (length/diameter ratio) and the determination of the diameter range of the fibrillated cellulose using microscopy images, it is possible to calculate an average length of the fibrillated cellulose, as long as the samples are homogeneous, and avoiding the difficulties of the entanglement of the fibers in the measure of their length.

(3) $$Aspect\;Ratio,\;AR\;\left(CN\right)=5.98\cdot \sqrt{\frac{1000}{Gel\;point,\;\emptyset_{g}\left(\frac{kg}{m^{3}}\right)}}$$

(4) $$Aspect\;Ratio,\;AR\;\left(EMT\right)=3.61\cdot {\left(\frac{1000}{Gel\;point,\;\emptyset_{g}\left(\frac{kg}{m^{3}}\right)}\right)}^{0.567}$$

To verify that the interval of C_o,opt_ previously indicated (0.5·C_o, opt,_ 1.5·C_o, opt_) in which a chosen C_o_ is closed to the C_o, opt,_ not requiring a second sedimentation experiment, eight different fibrillated celluloses with different Ø_g_ were tested at different C_o_. Sheet 5 of Excel Data Base includes the initial values of the sedimentation experiments. Table 4 shows the Ø_g_ of the fibrillated celluloses using the smoothing spline with an y-interception <0.0015·Ø_g_ and the AR using the CN and EMT theory. Then, with the C_o,opt_ of each sample (calculated as Example 1) the estimated Ø_g_ and AR are calculated and compared with the original. It is possible to observe that CN and EMT theories, previously developed by other authors, show differences around 10% in AR when the AR is up to 80, whereas these differences are bigger in shorter AR fibers. However, the AR compared among the Ø_g_ and estimated Ø_g_ are scarce and similar, not being the theory chosen to calculate the AR a relevant parameter.

**Table 4:** Gel point (Ø_g_) and AR.

Fibrillated cellulose sample (Nº)	1	2	3	4	5	6	7	8
Ø_g_ (kg/m^3^)	317	31.2	2.31	1.52	2.84	4.00	2.02	25.5
AR (CN)	10.6	33.9	124	153	112	95	133	37.4
AR (EMT)	7.0	25.8	113	143	100	83	122	28.9
C_o,opt_	18.1*	1.77	0.121	0.074	0.154	0.217	0.101	1.45
Estimated Ø_g_ (kg/m^3^)								
(Error,%)	Out of range*	30.3 (2.9%)	2.36 (2.2%)	1.60 (5.3%)	2.91 (2.5%)	4.22 (5.5%)	2.14 (5.9%)	25.6 (0.4%)
Estimated AR (CN) (Error,%)	Out of Range*	34.3 (1.3%)	123 (1.1%)	149 (2.9%)	111 (1.1%)	92 (2.7%)	129 (3.0%)	37.3 (0.4%)
Estimated AR (EMT) (Error,%)	Out of Range*	26.2 (1.7%)	111 (1.2%)	139 (2.9%)	99 (1.4%)	80 (3.0%)	118 (3.2%)	28.8 (0.2%)

⁎ As indicated in the limitations, this value in the equations of Table 2 is out of range due to the AR is under 20–30.

Finally, Table 5 shows the Ø_g_ and the AR using Eq. (2) with initial concentrations that are in the defined interval in which it is not necessary a second sedimentation experiment (0.5·C_o, opt_, 1.5·C_o, opt_). It is possible to observe that the error is under 7% for all Ø_g_ calculated and under 3% for the AR. Independently the theory used to calculate the AR, these errors are in the same order. On the other hand, when C_o_ is out of the mentioned interval, in some cases we obtain errors above the 7 and 3%, respectively.

**Table 5:** Gel point (Ø_g_) and AR calculated from C_o_ in and out of the interval (0.5·C_o, opt_, 1.5·C_o, opt_).

Fibrillated cellulose	1	2	3	4	5	6	7	8
C_o_ in the interval (0.5·C_o, opt_, 1.5·C_o, opt_)								
C_o_	Out of Range*	0.97	0.15	0.074	0.15/0.25	0.15/0.25	0.10	1.16/1.43
Ø_g_ (kg/m^3^) Error (%)		30.7 (1.7%)	2.33 (0.9%)	1.60 (5.5%)	2.78/2.99 (2.1/5.3%)	3.95/4.17 (1.3/4.3%)	2.03 (0.8%)	25.4/25.7 (0.2/1.0%)
AR (CN) Error (%)		34.1 (0.7%)	124 (0.2%)	149 (2.6%)	113/109 (0.9/2.7%)	95/93 (0.7/1.6%)	133 (0.4%)	37.5/37.3 (0.2/0.4%)
AR (EMT) Error (%)		26.0 (0.9%)	112 (0.5%)	139 (2.9%)	102/98 (1.2/2.8%)	83/81 (0.7/2.3%)	121 (0.3%)	29.0/28.8 (0.2/0.4%)
C_o_ out of the interval (0.5·C_o, opt_, 1.5·C_o, opt_)								
C_o_	1.5/3	0.5/3	0.25	0.15	0.75	0.75	0.05/0.25	0.11
Ø_g_ (kg/m^3^) Error (%)	311/308 (1.9/2.8%)	28.1/31.5 (9.9/0.9%)	2.50 (8.2%)	1.69 (11%)	3.54 (25%)	5.28 (32%)	1.87/2.36 (7.4/17%)	28.7 (13%)
AR (CN) Error (%)	10.7/10.8 (0.9/1.8%)	35.7/33.7 (5.2/0.6%)	120 (2.9%)	145 (5.0%)	101 (10%)	82 (13.2%)	138/123 (3.8/7.6%)	35.3 (5.8%)
AR (EMT) Error (%)	7.0/7.0 (1.1/1.6%)	27.4/25.6 (6.1/0.5%)	108 (4.4%)	135 (5.8%)	89 (11.7%)	71 (14.6%)	127/111 (4.5/8.4%)	27.0 (6.5%)

## Limitations

In general, gel point methodology is limited, due to the difficulties in sedimentation, in materials with AR under 20–30, which indicate that is useful only for fibrillated cellulose and not for crystalline cellulose materials such as cellulose nanocrystals or cellulose microcrystals.

The equation used to recalculate the optimal initial concentration (Table 2, Equation y-interception <0.0015·Ø_g_) is limited for cellulose materials with AR below 20–30. Below this value, the optimal initial concentration suggested can be above the gel point concentration and not settle, requiring lower initial concentrations than expected.

## Ethics Statement

Authors confirms that there is no ethical conflict.

## CRediT authorship contribution statement

**J.L. Sanchez-Salvador:** Methodology, Investigation, Writing – original draft. **M.C. Monte:** Supervision, Data curation. **C. Negro:** Writing – review & editing, Funding acquisition. **W. Batchelor:** Conceptualization, Funding acquisition. **G. Garnier:** Conceptualization, Funding acquisition, Supervision. **A. Blanco:** Investigation, Writing – review & editing, Project administration.

## Data Availability

Dataset for the simplification of gel point in fibrillated celluloses (Original data) (Mendeley Data)
